# Translocation of particles and inflammatory responses after exposure to fine particles and nanoparticles in an epithelial airway model

**DOI:** 10.1186/1743-8977-4-9

**Published:** 2007-09-25

**Authors:** Barbara Rothen-Rutishauser, Christian Mühlfeld, Fabian Blank, Claudia Musso, Peter Gehr

**Affiliations:** 1 Institute for Anatomy, Division of Histology, University of Bern, Bern, Switzerland

## Abstract

**Background:**

Experimental studies provide evidence that inhaled nanoparticles may translocate over the airspace epithelium and cause increased cellular inflammation. Little is known, however, about the dependence of particle size or material on translocation characteristics, inflammatory response and intracellular localization.

**Results:**

Using a triple cell co-culture model of the human airway wall composed of epithelial cells, macrophages and dendritic cells we quantified the entering of fine (1 μm) and nano-sized (0.078 μm) polystyrene particles by laser scanning microscopy. The number distribution of particles within the cell types was significantly different between fine and nano-sized particles suggesting different translocation characteristics. Analysis of the intracellular localization of gold (0.025 μm) and titanium dioxide (0.02–0.03 μm) nanoparticles by energy filtering transmission electron microscopy showed differences in intracellular localization depending on particle composition. Titanium dioxide nanoparticles were detected as single particles without membranes as well as in membrane-bound agglomerations. Gold nanoparticles were found inside the cells as free particles only. The potential of the different particle types (different sizes and different materials) to induce a cellular response was determined by measurements of the tumour necrosis factor-α in the supernatants. We measured a 2–3 fold increase of tumour necrosis factor-α in the supernatants after applying 1 μm polystyrene particles, gold nanoparticles, but not with polystyrene and titanium dioxide nanoparticles.

**Conclusion:**

Quantitative laser scanning microscopy provided evidence that the translocation and entering characteristics of particles are size-dependent. Energy filtering transmission electron microscopy showed that the intracellular localization of nanoparticles depends on the particle material. Both particle size and material affect the cellular responses to particle exposure as measured by the generation of tumour necrosis factor-α.

## Background

Besides the generation of ultrafine particles from combustion processes (UFP), an increasing number of manufactured nanoparticles (NP), defined as structures with a diameter of 1–100 nm, are released into air, water and soil [1,2]. Manufactured NP have many novel applications, thus furthering the progress of nanotechnology [3]. One aim of nanotechnology is to deliver therapeutic and diagnostic agents and is referred to as nanomedicine [4]. NP are already present in many products, such as suncream, other cosmetics or leisure wear [5], and human exposure to NP is strongly increasing.

Upon inhalation, airborne UFP or NP come into contact with a series of structural and functional barriers that protect the respiratory system against harmful and innocuous particulate material [6]. This is important as the internal surface area of the lungs is vast (alveoli and airways approximately 140 m^2^) [7] facilitating efficient access to the lung tissue. However, despite the existence of these barriers, respiratory diseases related to inhalation of airborne UFP are frequent and increasing [8-10]. The physiological barriers of the respiratory system may not be effective to protect the body from particles < 0.1 μm in size. Deposition as well as the subsequent fate of inhaled UFP and NP is different from that of larger particles. It has been shown that titanium dioxide(TiO_2_) particles with a diameter of less than 0.1 μm are able to cross cellular membranes in a rat lung exposure model that did not involve commonly known phagocytotic mechanisms [11], and that a small fraction of TiO_2 _NP are rapidly transported from the airway lumen to the connective tissue and subsequently released into the systemic circulation [12]. As these particles were also found inside pulmonary capillary erythrocytes it is not surprising that in other studies UFP could be localized in many other organs of the body, including the liver, the heart and the nervous system within a few hours after deposition in the respiratory system [13-15]. Once inside the organism ambient particulate matter may cause adverse health effects due to increased pulmonary and cardiovascular morbidity as shown by a number of epidemiological studies [8,10,16,17]. In this context, a specific toxicological effect has been attributed to UFP recently [18]. It has been described that inhaled combustion-derived UFP provoke oxidative stress causing inflammation as well as oxidative adducts in the epithelium that may contribute to carcinogenesis [19]. A growing body of literature supports the concept that manufactured NP share the toxic potential of UFP and it is generally accepted that the toxicity of NP depends on a variety of their properties [20,21], such as size [22], bulk material, surface charge [23].

NP have the capacity to enter different cell types and evade endocytotic pathways [11,24,25]. *In vitro *experiments revealed penetration of NP into mitochondria of macrophages and epithelial cells, associated with oxidative stress and mitochondrial damage [26]. In addition, penetration of NP into the nucleus has been shown in a number of studies [11,27-29]. Once inside the cells, NP may cause several biological responses including the enhanced expression of pro-inflammatory cytokines [30], the generation of reactive oxygen species [31] and DNA strand breaks [32]. Further associations between oxidative stress and inflammation responses are described in the literature [19,33,34] and inflammation responses are associated again with adverse health effects [35,36].

In order to determine the importance of particle size and material on the translocation behaviour of NP and their potential to induce cellular responses we have used a triple cell co-culture model of the airway wall composed of monocyte derived macrophages (MDM), epithelial cells and monocyte derived dendritic cells (MDDC) [37]. First, the intracellular localization of fluorescently labeled polystyrene fine particles (1 μm) and NP (0.078 μm) within the different cell types was analyzed by laser scanning microscopy (LSM) combined with digital image restoration. The quantitative distribution of the different particles among the different cell types was compared using a contingency table analysis. Second, the intracellular localization of NP made of different materials (gold, and TiO_2_) was studied using energy filtering transmission electron microscopy (EFTEM). TiO_2 _NP were among the earliest industrially produced and applied NP in everyday applications [5]. Gold NP are in use for optical, electronic, magnetic, catalytic, and biomedical applications [38]. Finally, the potential of the particles to induce a pro-inflammatory response in dependence on size and material was investigated by measurement of the tumor necrosis factor alpha (TNF-α).

## Results

### Translocation of polystyrene fine particles and nanoparticles

**Figure 1:**
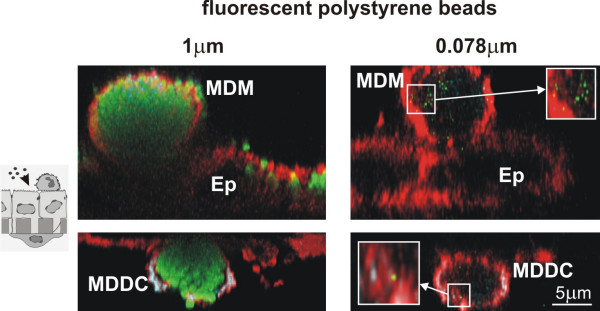
Intracellular particle localisation in tripe cell co-cultures visualised by LSM. Cells at the upper side of the insert (upper row) were stained for CD14 (MDM, turquoise) and F-Actin (all cells, red); cells at the lower side (lower row) for CD86 (MDDC, turquoise) and F-Actin (all cells, red). Fluorescently labelled polystyrene particles (1 μm, and 0.078 μm) are shown in green. MDM and MDDC were filled with particles, considerably fewer particles were found in epithelial cells (Ep). All images represent xz-projections.

In order to compare the entering and translocation of fine particles and NP into cells of the airway epithelial barrier the interaction of fluorescently labelled polystyrene particles of two different sizes (1 μm, and 0.078 μm) with the cells of the triple cell co-culture system was studied by LSM in combination with immunofluorescence methods and digital image restoration. After incubation with particles for 24 h, cells were fixed and stained for F-Actin in addition to the labelling of specific surface markers, CD14 for MDM, and CD86 for MDDC. MDM and MDDC were filled with 1 μm particles, and only few of these particles were detected in epithelial cells (Fig. 1). By applying a deconvolution algorithm even NP, i.e. 0.078 μm polystyrene particles, could be visualized and we found many particles in MDM as well as in MDDC, and again only few particles in epithelial cells (Fig. 1).

Special attention was paid to the integrity of the epithelial layer. Transepithelial electrical resistance (TEER) measurements were performed before and at various times during incubation with the particles. Addition of particles did not influence the integrity of the tight junctions when compared to control cultures and TEER values within a range from 140 to 190 Ωcm^2 ^in the control cultures and in cultures treated with particles were measured.

### Quantitative analysis of intracellular polystyrene fine particles and nanoparticles

**Figure 2:**
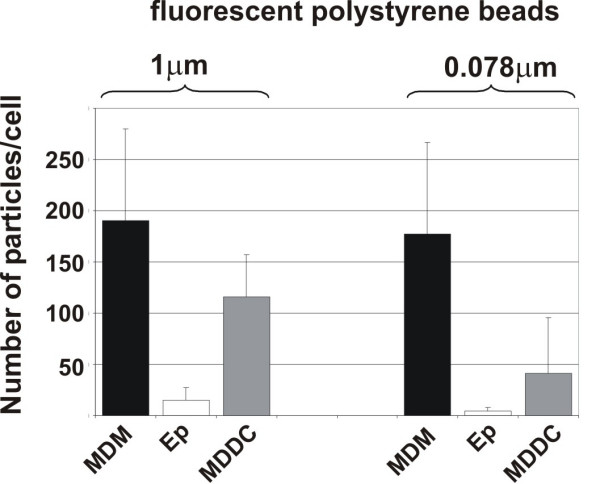
Quantification of particles inside individual cells. Intracellular particle numbers were analysed in cultures exposed to 1 μm, and 0.078 μm polystyrene particles with the software Diacount. Epithelial cells (Ep). Number of intracellular particles was counted in individual cells. Data are expressed as the mean of 3–4 experiments (10–14 cells scanned per experiment by LSM).

Since we had seen a qualitative difference in the number of intracellular particles within the different cell types, the number of fluorescently labeled polystyrene particles of different sizes (1 μm, and 0.078 μm) was counted using a specific software, called "DiaCount", which allows to precisely and reliably count thousands of objects in 3D image stacks created by LSM. As for 1 μm particles we found 190 ± 90 (SD) particles in MDM, 116 ± 41 particles in MDDC, and 15 ± 12 particles in epithelial cells (Fig. 2). For 0.078 μm particles we found: 177 ± 90 particles in MDM, 41 ± 55 particles in MDDC, and 4 ± 4 particles in epithelial cells (Fig. 2).

### Contingency table analysis for polystyrene fine particles and nanoparticles (Table 1)

**Table 1:** Contingency table analysis of particle numbers in different cell types among the particle sizes

**Cell type**	**P_obs_(P_exp_) 1 μm**	**P_obs_(P_exp_) 0.078 μm**	**Row total**	**Chi^2^**
AM	190 (216.7)	176.67 (149.97)	366.67	**3.29; 4.75**
Epithelial cells	14.69 (11.3)	4.43 (7.82)	19.12	1.017; 1.47
DC	116.22 (92.9)	41 (64.31)	157.22	**5.85; 8.45**
Column total	320.91 (320.91)	222.1 (222.1)	543.01	**24.827**

In the first column, the different cell types, i.e. MDM, epithelial cells, or MDDC, are denoted. In each of the cell types, the mean number of particles was counted (P_obs_). According to the equation given in the methods section the number of expected particles was calculated (P_exp_). To see whether the distribution of counted particles within the three cell types was different for the different particle sizes, partial chi^2 ^values and total chi^2 ^were calculated. For 2 degrees of freedom (3-1 cell types x 2-1 particle sizes) and with a total chi-squared value of 24.827 the null-hypothesis that the differently sized particles are similarly distributed among the different cells has to be rejected (p < 0.01). Those cell types and particle sizes that make a substantial contribution to the total chi-squared (more than 10%) are highlighted in bold letters within the table.

With 3-1 compartments (cell types) times 2-1 groups (differently sized particles) = 2 degrees of freedom, and a total chi squared value of 24.83 the null-hypothesis that the differently sized particles are similarly distributed among the different cells has to be rejected (p < 0.01). The partial chi-squared value contributed 10% or more to the total chi-squared for both particle sizes in macrophages and MDDC. For 1 μm particles there were fewer particles than expected in macrophages and more particles than expected in MDDC. In contrast, for 0.078 μm particles, there were more particles than expected in MDM and fewer particles than expected in MDDC.

### Intracellular localisation of differently composed nanoparticles

In order to analyse whether the material of the NP determines its intracellular localization, TiO_2 _NP(mean diameter of 0.032 μm) and colloidal gold NP (diameter of 0.025 μm) were visualized and analysed in cells using EFTEM [25]

**Figure 3:**
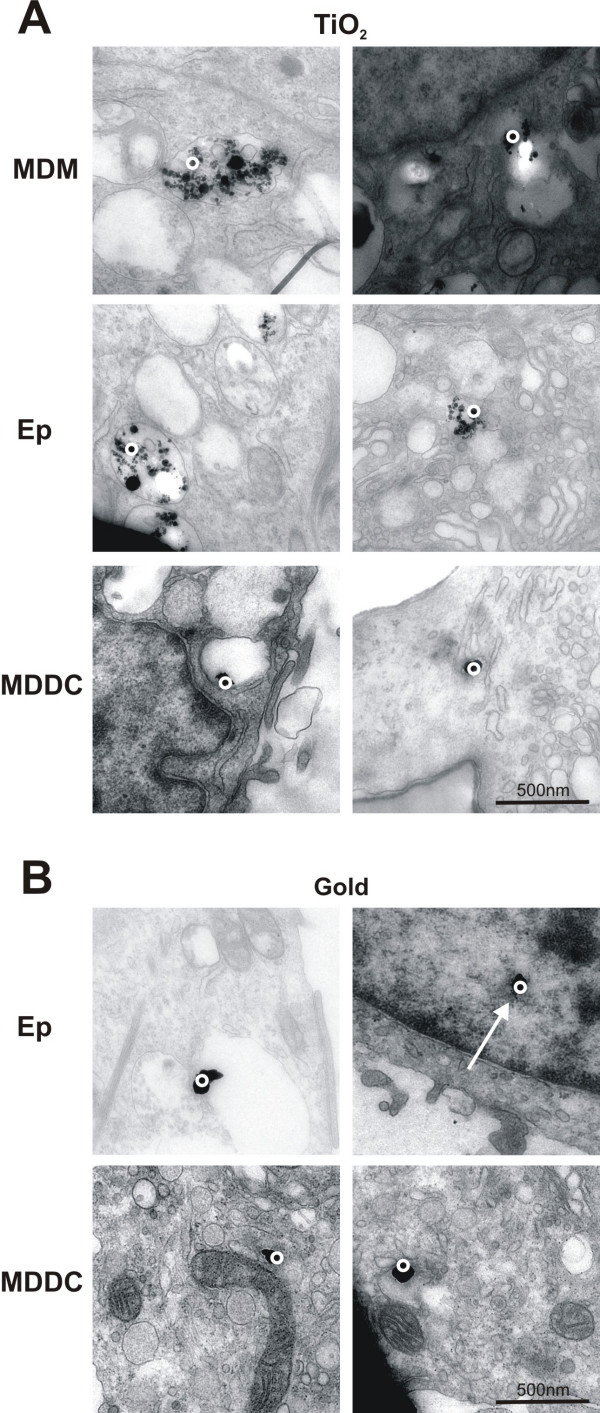
EELS images of cells containing TiO_2 _and silver enhanced gold particles. TiO_2 _particles (A) were found inside all cell types, i.e. MDM, epithelial cells, and MDDC, as aggregates in vesicles (A, left panel), and as single particles or as small aggregates free in the cytoplasm (A, right panel). Silver enhanced gold particles (B) were found in all three cell types as single particles or as small aggregates only free in the cytoplasm and even in the nucleus (B, arrow). The circles mark the region where the element analysis was performed.

Bigger membrane-bound aggregates (>0.2 μm) of TiO_2 _NP were identified by analytical TEM in all cell types, i.e. MDM, epithelial cells and MDDC (Fig. 3A). In addition we found single particles and smaller (<0.2 μm) aggregates that were not membrane bound (Fig. 3A). Interestingly, silver enhanced gold particles were not observed in vesicles they were only detected as single particles or as small aggregates (<0.1 μm) free in the cytoplasm (Fig. 3B). Some gold particles were even found in the cell nucleus (Fig. 3B arrow).

### Influence of particle size and composition on the inflammatory potential

**Figure 4:**
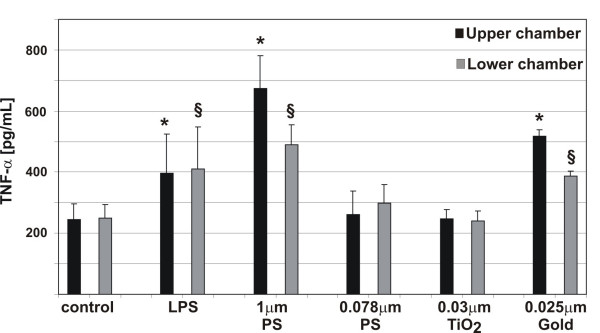
TNF-α release in triple cell co-cultures upon particle incubation. TNF-α levels in the supernatants (upper chamber, lower chamber) were measured by ELISA. TNF-α release in cells exposed to LPS, 1 μm, and 0.078 μm polystyrene particles, TiO_2_, and gold NP. Values are means ± SD of 3 experiments. * indicates a statistical difference to the levels in the supernatants in the control of the upper chamber, ^§ ^indicates a statistical difference to the levels in the supernatants in the control of the lower chamber.

As shown above, the morphological examination of the particle-cell interactions showed that all particle types used in this study are able to enter the different cells of the co-culture system. Therefore, we determined the pro-inflammatory cytokine TNF-α in the culture supernatants after incubations with particles for 24 h (Fig. 4). In the control cultures minor TNF-α concentrations were measured. When LPS (lipopolysaccaride, a positive control) and 1 μm particles were added to the cell cultures, the TNF-α signal increased significantly. No increase in TNF-α concentration was seen when 0.078 μm polystyrene particlesor TiO_2 _NP were added to the cultures. We found a significant increase of TNF-α in the supernatants after applying gold NP.

## Discussion

The understanding of the possible functional and pathological disorders induced in the respiratory tract by NP requires the investigation of the direct effects of these particles on state and activity of lung cells. Therefore, we have used our established *in vitro *model of the airway wall [37] to compare the translocation behaviors of fine particles and NP, to study the intracellular localization of NP and to investigate the potential of the particles in dependence on size and material to induce a pro-inflammatory response.

First, fine fluorescently labeled polystyrene particles (1 μm) and NP (0.078 μm) have been added to the cells and visualized by LSM combined with digital image restoration [25]. Both particle sizes were found in all three cell types, however, the number of particles was always considerably smaller in the epithelial cells than in both other cell types. Quantitative analysis revealed that the number of 1 μm and 0.078 μm particles inside MDM was twice the number found in MDDC. This finding confirms what was reported by Kiama et al. [39] who showed that MDM are twice as phagocytic as immature MDDC *in vitro*. Only a small particle number was found inside epithelial cells for both particle sizes. The quantitative analysis for the NP distribution in the different cell types was performed as described by Mühlfeld et al. [40]. One main conclusion that can be drawn from this analysis is that the distribution of particles within the different cell types is not equal among the different particle sizes. The difference is characterized by a significant lower number of NP to be localized in the MDDC in comparison to the larger particles. Conversely, the number of particles localized in MDM was higher than expected for 0.078 μm NP and lower than expected for the larger particles. Recently, we have shown that MDDC and MDM collaborate as sentinels against fine particles by building a transepithelial interdigitating network of cell processes [41], so the current data underline that fine particles might actively be transported from MDM to MDDC, whereas the nano-sized material has different translocation characteristics. It is tempting to speculate that the unique entering mechanisms of NP may prevent the physiological interplay between macrophages and dendritic cells to a certain degree. Concluding from our results and from earlier published findings [11,25] we confirmed the phagocytic uptake for 1 μm particles whereas particles < 0.1 μm may have the property of entering cells by unknown mechanisms (what we called adhesive interaction).

All polystyrene particle types we used in this study were observed in MDDC in the triple cell co-culture system, although the MDDC, residing underneath the epithelial monolayer, were thought not to be directly exposed to particles. Since the TEER did not decrease during the experiment we assume that the tight junctions were not opened after addition of particles to the medium. However, our studies showed that MDDC formed fine cytoplasmic processes towards the luminal side or even migrated as complete cells to the apical side of the epithelial barrier wall to take up particles of a diameter of 1 μm deposited on the epithelial apical surface [41]. However, it was the tightness of the epithelium which influenced the migration index of the MDDC, but not the mechanism of particle uptake [41]. Whether the cells are interacting in a similar manner after exposure of the cultures to NP is the aim of further investigations.

In order to draw any conclusions whether NP induce cellular responses it is indispensable to know if particles are attached to the cell membrane or have entered the cells. Sophisticated microscopic methods have been applied for the visualization of manufactured NP as described in Rothen-Rutishauser et al. [25]: LSM in combination with image restoration for fluorescently labeled polystyrene NP, and EFTEM for gold NP as well as for TiO_2 _NP. As shown in LSM and TEM micrographs, polystyrene, gold and TiO_2 _NP can enter cells, even the non-phagocytic A549 epithelial cells. This confirms previous reports that NP can enter many cell types [11,42], even non-phagocytic cells like red blood cells which have neither phagocytic receptors at the surface nor are they equipped with a phagocytic apparatus [25]. For some NP the cell membrane does not seem to exert a barrier function; it seems as if they just pass through the membrane. We have currently no idea how these particles penetrate through the cell membrane. The entering mechanisms are currently under debate. Some assume these particles to enter cells by any endocytic processes other suggest mechanisms different from endocytosis like diffusion, membrane fluidity, passing through channels or further by adhesive interactions as for instance electrostatic forces, Van der Waals- or steric interactions [11,43,44]. Clearly, the entering mechanisms of NP need to be further investigated.

TiO_2 _was found inside all three cell types of the triple cell co-culture model, i.e. MDM, epithelial cells, and MDDC, as membrane-bound larger aggregates as well as free as smaller aggregates or individual particles in the cytoplasm. Other studies have shown vesicles containing mostly large aggregates of TiO_2 _in A549 cells [45] or membrane-bound ceria NP agglomerates in human lung fibroblasts [46]. However, in all these studies only conventional TEM was used to detect the particles, and it might well be that single NP or small aggregates of few particles could not be identified. In our study gold NP were detected only free in the cytoplasm of all cell types. These findings are in contrast to the study from Takenaka et al. [47] who detected gold NP aggregates inside small vesicles in macrophages of the rat lung. It is not clear why we could not detect vesicle containing gold particles, however, it might be that in the study from Takenaka et al. [47] single NP or small aggregates were not detectable by conventional TEM in the cytoplasm. We also found gold particles inside the cell nuclei. Penetration of NP into the nucleus has been shown in a number of studies [11,27-29]. Tsoli and colleagues[28] have shown that 1.4 nm Au_55 _particle clusters interact with DNA in a way which may be the reason for the strong toxicity of these tiny particles towards human cancer, since it is generally known that DNA double-strand breaks may cause cancer [48]. Of great interest is the stereological analysis of TiO_2 _and gold NP, which is planned in future studies.

A crucial reaction of cells to particles deposited in the lung is the release of cytokines from activated epithelial cells, MDM and MDDC. The essential role of the pro-inflammatory chemokine TNF-α in relation to lung injuries caused by ambient UFP or manufactured NP was described by Donaldson and colleagues [19]. In our studies TNF-α release after exposure to 1 μm polystyrene particles was significantly higher than in cultures exposed to 0.078 μm particles. Moreover, varying responses have been found with different types of NP, like polystyrene, gold, and TiO_2 _particles. Interestingly, only the exposure to gold NP was found to induce a pro-inflammatory response.

In conclusion, all particle types were detected inside the three cells of the triple cell co-culture model independent of their composing material and size. However, the distribution of fine and nano-sized polystyrene particles among the cells of the culture system was different suggesting size to be an important factor determining the translocation characteristics of particles. Additionally, the intracellular localization differed between TiO_2 _and gold NP suggesting different modes of intracellular trafficking and, possibly, entering mechanisms depending on the particle material. In accordance, both size and material of the particles affected the toxicological potential of the particles, i.e. the TNF-α generation. Whether the different inflammatory potential between differently composed NP is related to their entering mechanism and intracellular target structures needs further research.

## Methods

### Triple cell co- cultures

Cultures were prepared as previously described by Rothen-Rutishauser et al. [37]. Briefly, A549 cells (passage 10–40) were grown on cell culture inserts (surface area of 4.2 cm^2^, pores of 3.0 μm in diameter, high pore density PET membranes for 6-er well plates; (BD Biosciences, Basel, Switzerland). MDM and MDDC were derived from human blood monocytes as already described [37]. Briefly, peripheral blood monocytes were isolated from buffy coats (blood donation service, Berne, Switzerland) and cultured in the same medium as used for the epithelial cells except for the supplementation of 5% human serum (blood donation service Bern, Switzerland) instead of 10% foetal calf serum. For the generation of MDDC the monocytes were cultured for 7–10 d in medium supplemented with 34 ng/mL IL-4 (Sigma, Fluka Chemie GmbH, Buchs, Switzerland) and with 50 ng/mL GM-CSF (R&D Systems, Oxon, UK), whereas the MDM were obtained without any additional supplements for 7–10d. Epithelial cells were cultured for 7 days before MDM were added on top of the epithelial monolayer and MDDC underneath the insert membrane. The triple cell co-cultures were kept overnight in medium supplemented with 1% L-Glutamine, 1% penicillin/streptomycin, and 5% heat inactivated (pooled) human serum at 37°C in 5% CO_2 _humidified atmosphere.

### Particle incubation

Commercially available particles were used: Fluoresbrite™ plain yellow green microspheres with diameters of 1 μm (Polysciences, Chemie Brunschwig AG, Basel, Switzerland); Fluorescent particles, yellow green with a mean diameter of 0.078 μm (KiskerGbR, Chemie Brunschwig AG, Basel, Switzerland); Bovine Serum Albumin (BSA) gold tracers with a diameter of 0.025 μm (Aurion, Anawa Trading SA, Wangen, Switzerland); Titanium (IV) oxide (TiO_2_), anatase 99.9% (metal basis)) with a mean diameter of 0.02–0.03 μm (Alfa Aesar, Johnson Matthey GmbH, Karlsruhe, Germany).

All particle dilutions were sonicated for 2 min prior to incubation with cells in order to avoid aggregation. Polystyrene particles were diluted in RPMI 1640 medium and adjusted to a concentration of 10^10 ^particles/mL. As recommended from the company gold particles were first dialyzed in PBS for 24 hours to remove the sodium acid and were diluted in RPMI 1640 to a final dilution of 10^10 ^particles/mL. A stock solution of TiO_2 _particles in millipore water (2.5 mg/mL) was diluted in RPMI 1640 to a final concentration of 5 μg/mL. One mL of this suspension was then added to cell cultures. Incubations were done for 24 h before analysis of the cells. Each experiment was repeated between 3 to 5 times.

### TEER measurements

TEER was measured with the Millicell-ERS system (MERS 000 01, Millipore AG, Volketswil, Switzerland). The mean of 3 measurements per insert was determined. The electrical resistance of filters without cells was subtracted from all samples and the resistance values were multiplied with the surface area of the inserts (4.2 cm^2^). Electrical resistance was measured in triple cultures to follow the epithelial tightness as described in Rothen-Rutishauser et al. [49].

### Cell labeling and fixation

Cells were washed in phosphate buffered saline (PBS, 10 mM, pH 7.4: 130 mM NaCl, Na_2_HPO_4_, KH_2_PO_4_) and fixed for 15 min. at room temperature in 3% paraformaldehyde in PBS. Fixed cells were treated with 0.1 M glycine in PBS for 5 min. and permeabilized in 0.2% Triton X-100 in PBS for 15 min. The cells were incubated with the first and second antibodies for 60 min each at room temperature. Preparations were mounted in PBS:glycerol (2:1) containing 170 mg/mL Mowiol 4–88 (Calbiochem, VWR International AG).

Antibodies were diluted in PBS as follows: mouse anti-human CD14 1:20 (Clone UCHM-1, C 7673, Sigma), mouse anti-human CD86 1:20 (Clone HB15e, 36931A, PharMingen, BD Biosciences,), goat anti-mouse cyanine 5 1:50 (AP124S, Chemicon, VWR International AG, Life Sciences, Lucerne, Switzerland), and phalloidin rhodamine 1:100 (R-415, Molecular Probes, Invitrogen AG, Basel, Switzerland).

### Tumor necrosis factor alpha detection

Following particle incubation, supernatants from triple cell co-cultures in the upper and lower chamber were collected separately and stored at -70°C. After centrifugation, TNF-α was quantified by a commercially available DuoSet ELISA Development kit (R&D Systems, Catalogue Number: DY 210, Oxon, UK) according to the manufacturer's recommendations. The assay was repeated twice, each in duplicates. Between three to five experiments were carried out for each combination of particles and cell types. The incubation time of the cells with the particle suspension was 24 h.

The mouse anti-human TNF-α capture antibody was coated overnight in 96-well immunoassay plates (NUNC, MaxiSorp) at a concentration of 4 μg/mL in PBS at room temperature. Differing to the producer's protocol the plate was blocked with PBS supplemented with 1% BSA, 5% sucrose and 0.05% sodium acid for 1 h at room temperature (as opposed to the reagent dilution provided with the test kit). After washing with buffer, supernatants from samples and the standards (recombinant human TNF-α, concentrations from 0.02 to 10 ng/mL of TNF-α) were pipetted into the wells and incubated at room temperature for 2 h. After washing, biotinylated goat anti-human TNF-α detection antibodies were added to the wells and incubated for 2 h at room temperature. After washing, horseradish peroxidase conjugated streptavidin was added to the plates, which were then incubated for 20 min at room temperature in the dark. Finally, the substrate solution (Tetramethylbenzidine/H_2_O_2 _(R&D Systems, Art.Nr. DY999)) was added. After 20 min in darkness, the colour development was stopped by 2NH_2_SO_4 _and the plate was put on the shaker for 10 min (differing from the protocol). Then the absorbance was read at 450 nm using an ELISA reader (SpectraMax 340 PC or Benchmark Plus Microplate Spectrophotometer (BioRad, Hempel Hempstead, UK)). The concentration of TNF-α was determined by comparing the absorbance of the samples with standard recombinant human TNF-α and calculated with the Office Excel program from Microsoft.

### Laser scanning microscopy and image restoration

A Zeiss LSM 510 Meta with an inverted Zeiss microscope (Axiovert 200 M, Lasers: HeNe 633 nm, HeNe 543 nm, and Ar 488 nm) was used. Image processing and visualization was performed using IMARIS, a 3D multi-channel image processing software for confocal microscopic images (Bitplane AG, Zurich, Switzerland). For the localization and visualization of particles at high resolution a deconvolution algorithm was applied [25] using the Huygens 2 software (Scientific Volume Imaging B. V., Hilversum, Netherlands) in order to increase axial and lateral resolutions and to decrease noise.

### Particle quantification

After the image acquisition the total particle number in the scans was counted with the particle tracking software Diacount (Semasopht, Lausanne, Switzerland; ). For each experimental sample cells were randomly scanned with LSM. Experiments were performed in triplicates or quadruplicates, and 10–15 cells were scanned for each data point. The particles were counted within individually defined cell types, which were labelled for specific cell surface markers (CD14 for MDM, and CD86 for MDDC, F-Actin for the epithelial cells).

### Energy filtering transmission electron microscopy

EFTEM analysis was performed as described in Rothen-Rutishauser et al. [25]. Briefly, cells were fixed with 2.5% glutaraldehyde in 0.03 M potassium phosphate buffer, pH 7.4. The cells were postfixed with 1% osmium tetroxide in 0.1 M sodium cacodylate buffer, and with 0.5% uranyl acetate in 0.05 M maleate buffer. Cells were then dehydrated in a graded series of ethanol and embedded in Epon. Ultrathin (≤ 50 nm) sections were cut, mounted onto uncoated 600-mesh copper grids, and stained with uranyl acetate and lead citrate. The presence and localization of TiO_2 _particles was investigated with a LEO 912 transmission electron microscope (Zeiss, Oberkochen, Germany) using electron energy loss spectroscopy. Since gold was not suitable for elemental analysis the gold particles were coated with silver using silver enhancement reagent (AURION R-GENT SE-LM; Aurion, Anawa trading SA).

### Statistics

The results of the TEER and the ELISA measurements are expressed as mean values with the standard deviation of the mean (SD). The statistical analysis was performed using SigmaStat for Windows (Version 3.10, Systat Software, Inc., Richmond, California, USA) statistical software. Two groups were compared using Student's t-Test. p < 0.05 was considered to be significant.

The distribution of the different particles among the different cell types was compared using a contingency table analysis as described by Mühlfeld et al. [40]. The observed numbers of polystyrene particles were compared by use of a contingency table analysis to depict changes in particle distribution among the different cell types according to the size group of the particles. From the number of observed particles, the number of expected particles was calculated according to the equation: column total x row total/grand total = expected number of particles. Partial chi-squared values for each cell type and particle size are given by: (observed particles – expected particles)^2^/expected particles = partial chi squared value. From the partial chi squared values the total chi squared value was calculated by summing up the partial values. The total chi squared value indicated whether the distributions of particles among the different cell types differed depending on particle size. The partial chi squared values helped to identify those compartments that contributed substantially to the different distributions. A convenient cut-off value for a substantial contribution is given by 10% or more of the total chi-squared.

## List of abbreviations

BSA Bovine serum albumin

EFTEM Energy filtering transmission electron microscopy

LSM Laser scanning microscopy

MDDC Monocyte derived dendritic cells

MDM Monocyte derived macrophages

NP Nanoparticles

PBS Phosphate buffered saline

TiO_2 _Titanium dioxide

TEM Transmission electron microscopy

TNF-α Tumour necrosis factor alpha

UFP Ultrafine particles

## Competing interests

The author(s) declare that they have no competing interests.

## Authors' contributions

**BRR **carried out the design of the study, has done the acquisition of the data, the analysis and interpretation of data, and drafted the manuscript. **ChM **has performed the contingency table analysis; he has been involved in the analysis and interpretation of data, as well as drafting the manuscript. **FB **has been involved in the analysis and interpretation of data, and in revising the manuscript critically for important intellectual content. **CM **has performed the ELISA measurements and has done the data analysis. **PG **was the project leader, he has intellectually accompanied the experimental work; he has been involved in revising the manuscript critically for important intellectual content and has given final approval of the version to be published.
